# A Feasibility Study for Implementation “Health Arcade”: A Study Protocol for Prototype of Multidomain Intervention Based on Gamification Technologies in Acutely Hospitalized Older Patients

**DOI:** 10.3390/ijerph17218058

**Published:** 2020-11-01

**Authors:** César Cuevas-Lara, Mikel Izquierdo, Fabiola Zambom-Ferraresi, Mikel L. Sáez de Asteasu, Itxaso Marín-Epelde, Chenhui Chenhuichen, Fabricio Zambom-Ferraresi, Robinson Ramírez-Vélez, Antonio García-Hermoso, Álvaro Casas-Herrero, Amaya Capón-Sáez, Lucia Lozano-Vicario, Irene Criado-Martín, Marina Sánchez-Latorre, Cristina Antoñanzas-Valencia, Nicolás Martínez-Velilla

**Affiliations:** 1 Navarrabiomed, Hospital Complex of Navarra (CHN)—Public University of Navarra (UPNA), Navarra Health Research Institute (IdisNa), Pamplona, C/Irunlarrea 3, 31008 Pamplona, Navarra, Spain; mikel.izquierdo@gmail.com (M.I.); fabiola.zambom.ferraresi@navarra.es (F.Z.-F.); mikel.lopez.saezdeasteasu@gmail.com (M.L.S.d.A.); fabricio.zambom.ferraresi@navarra.es (F.Z.-F.); robin640@hotmail.com (R.R.-V.); antonio.garciah@unavarra.es (A.G.-H.); cristina.antonanzas.valencia@navarra.es (C.A.-V.); 2 Geriatric Department, Hospital Complex of Navarra (CHN), C/Irunlarrea 3, 31008 Pamplona, Navarra, Spain; itxaso_m_1@hotmail.com (I.M.-E.); chenhuichen253@gmail.com (C.C.); alvaro.casas.herrero@navarra.es (Á.C.-H.); amaya_1927@hotmail.com (A.C.-S.); lucia.lozanovicario@gmail.com (L.L.-V.); irene.criado.martin@navarra.es (I.C.-M.); msanchezlatorre@gmail.com (M.S.-L.); 3 Center for Biomedical Research in Network on Healthy Fragility and Aging (CIBERfes), Carlos III Health Institute, 28029 Madrid, Spain; 4 Laboratory of Physical Activity, Sports and Health Sciences, University of Santiago de Chile (USACH), Santiago 71783-5, Chile

**Keywords:** serious games, mHealth, functional capacity, geriatric, aged, hospitalization

## Abstract

The aim of this article is to present the research protocol for a study that will evaluate the feasibility of implementation of Health Arcade prototype multidomain intervention based on physical and cognitive training using gamification technologies at improving care for older people hospitalized with an acute illness. A total of 40 older people will be recruited in a tertiary public hospital at Pamplona, Spain. The intervention duration will be four to nine consecutive days. Additionally, the patients will receive encouragement for maintaining active during hospital stay and for reducing sedentary time. Primary implementation-related outcomes will be the adherence to treatment (i.e., number of games and days completed during the intervention period), reaction or response time, and number of success and failures in each game per day. Secondary implementation-related outcomes will be self-perceived grade of difficulty, satisfaction, enjoyment per game and session, and self-perceived difficulties in handling the prototype hardware. Other health-related outcomes will also be assessed such as functional capacity in activities of daily living, mood status, quality of life, handgrip strength, physical activity levels, and mobility. The current study will provide additional evidence to support the implementation of multidomain interventions designed to target older persons with an acute illness based on friendly technology. The proposed intervention will increase accessibility of in-clinical geriatrics services, improve function, promote recovery of the health, and reduce economic costs.

## 1. Introduction

The risk of loss of independence and the likelihood of developing new disabilities that increases with the ageing process is one of the major issues in developed countries. Such loss of functional independence, coupled with a gradual impairment of cognitive and physical abilities, is associated with a greater chance of institutionalization and mortality [1,2]. In this context, the World Health Organization (WHO) published the first “World Report on Ageing and Health” [3] in 2015. This framework emphasizes that care for older people must shift their main focus from disease to intrinsic capacity (i.e., physical function). This becomes particularly important when dealing with old people at risk of disability, also known as frail, to act on them early on.

One of the major events leading to disability in elderly adults is usually acute medical illness with its subsequent hospitalization. Hospitalization-acquired disability incidence usually varies between 5% and 60% [4,5]. Recent evidence has shed light on hospitalization-related iatrogenic disability in elderly people, which is frequently characterized by lengthy periods of bedrest. Previous studies have observed that the time that older adults spend outside of said bed, pacing or walking along available spaces in the hospitals ranges from 7 to 43 min per day, reaching 83 min per day when also counting the time they spend on their feet [6,7]. This phenomenon has been named “pyjama paralysis” [8]. The decrease in mobility during hospital-stays in conjunction with the reduction of functional and physiological reserves in elderly hospitalized patients may result in many adverse events. These adverse events include loss of autonomy regarding activities of daily living (ADLs), a higher incidence of falls, cognitive impairment, sarcopenia, reduced caloric intake and social isolation [9]. Thus, the hospitalization should turn out to be the turning point and the appropriate place for establishing interventions to prevent hospitalization-acquired disability [10].

In this scenario, serious games could present an innovative intervention. Serious games are defined as games in which the final goal is to promote a change in the user, not the training in and of itself. This change could range between the acquiring of new knowledge to changes in attitudes or even changes in cognitive, physical, and/or social abilities [11]. The design of these games is based exclusively on virtual and technological environments, comprising an important component of virtual reality simulation. Furthermore, recent evidence has proposed the term “gamification,” which is used to mention the gaming elements in non-gaming contexts with the aim of reaching a behaviour change in the user [11,12,13,14]. These interventions have been proven to be effective in different settings, such as children, teenagers, or young adults, mainly in education, commerce, and communication. [15,16,17,18,19,20]. However, current evidence suggests it is still necessary to redesign and adapt these technologies to the characteristics and preferences of old patients in order to achieve maximum engagement [21].

**Figure 1:**
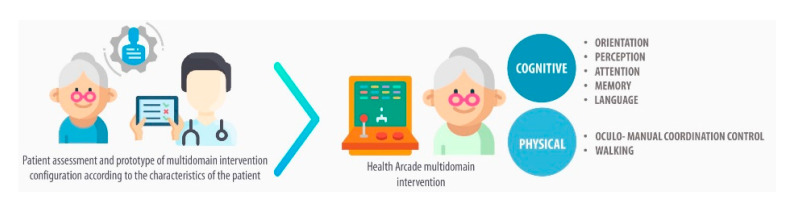
Health Arcade conceptual framework.

The Health Arcade project has designed to promote a change in geriatric patients’ routines during hospital stay by creating a favourable setting that will attract and motivate them for performing physical and cognitive activities to prevent iatrogenic nosocomial disability [22]. The main aim of this project is to validate the impact of a pilot-system of multidomain intervention (i.e., physical and cognitive training) using gamification technologies to improve patients’ functional abilities (Figure 1), while also encouraging personal motivation and internal locus of control, which have been shown to be predictive variables in health promotion [16,23,24].

## 2. Materials and Methods

### 2.1. Study Design and Setting

The Health Arcade utilized an uncontrolled, pre-post-test trial to determine the implementation of the prototype multidomain intervention based on physical and cognitive training using gamification technologies. This design was suitable for establishing the feasibility, appeal, and potential implementation of an innovative intervention prior to launching into a more costly and intensive randomized controlled trial design. The study will be implemented in the acute care for elderly (ACE) unit of the department of geriatrics in a tertiary public hospital (Hospital Complex of Navarre [CHN], Pamplona, Spain). This department is constituted by 35 beds allocated to the unit and a staff of 12 geriatricians. Admissions in the ACE unit proceed mainly from the accident and emergency department.

### 2.2. Study Participants and Eligibility Criteria

Older adults aged 75 or over admitted to the Department of Geriatrics of the CHN between September 2020 and January 2021. Inclusion and exclusion criteria are described in Table 1.

### 2.3. Participant-Selection and Consent Process

Participants will be identified during the geriatric assessment process at admission in the ACE unit. The geriatricians will conduct a screening interview to determine whether potentially eligible patients meet the inclusion criteria. Demographic characteristic (e.g., age, residence, and level of education, etc.) will be also included.

### 2.4. Ethics and Dissemination

The study followed the principles of the Declaration of Helsinki [25] and was approved by Hospital Complex of Navarre Clinical Research Ethics Committee on 6th November 2019 ID-PI_2019/96). All patients or their legal representatives provided written informed consent.

### 2.5. Interventions

**Figure 2:**
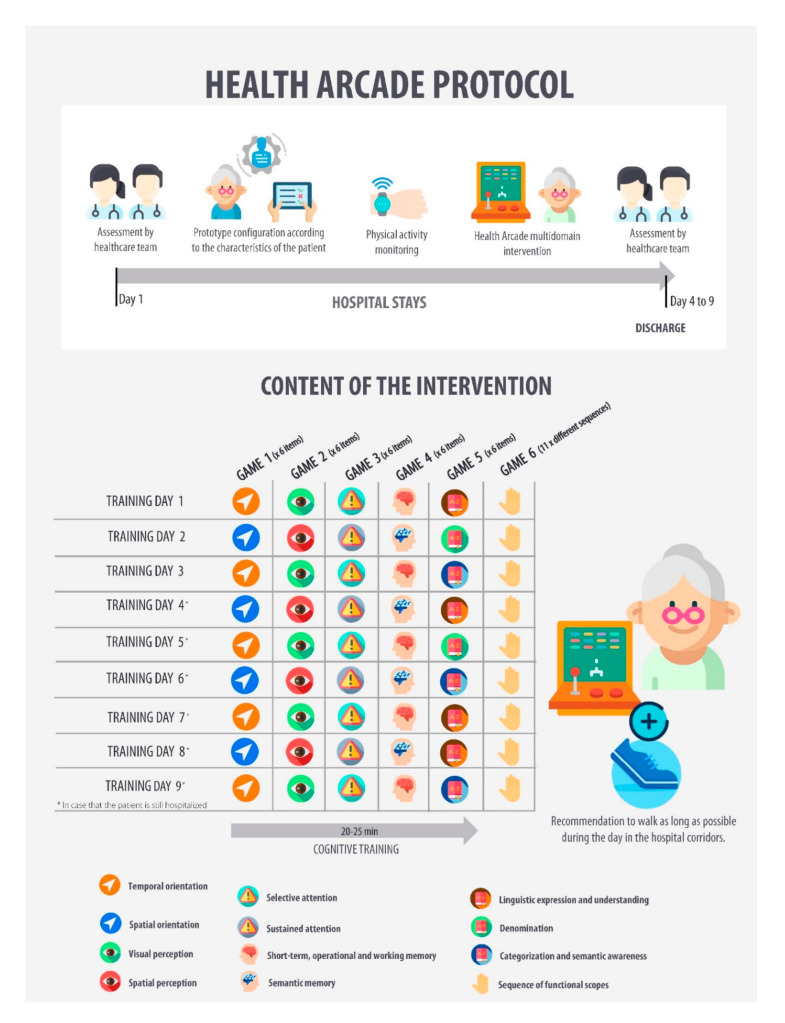
Health Arcade protocol.

The intervention will consist in a supervised training including cognitive and physical stimulation using the games of the Health Arcade prototype (developed by OUIPLAY sl., Dundee, UK). This pilot-system is made up of a selection of technologies, such as movement detection through Near-Field Communication Sensors, wireless communication systems, feedback systems (i.e., LEDs and screens), and game engine programmes for the use of virtual reality. The games will focus on different cognitive domains such as orientation, perception, attention span, working memory, language, and coordination. The intervention duration will be four to nine consecutive days. Additionally, the patients will receive encouragement for maintaining active during hospital stay and for reducing sedentary time. The details of the intervention are described in the Figure 2 and Table 2.

### 2.6. Outcomes Assessment

Feasibility will be considered in terms of the success of processes for recruiting and retaining participants and implementing the incentives program. The primary implementation-related outcome is the adherence to treatment (i.e., number of games and days completed during the intervention period), the reaction or response time and the number of success and failures in each game per day. These parameters are automatically recorded in real time by the Health Arcade prototype. We will consider as a measure of implementation success a score of at least 30 points per session. Secondary implementation-related outcomes are self-perceived grade of difficulty, satisfaction, enjoyment per game and session; perceived difficulties in handling the prototype hardware. These parameters are assessed by content validation questionnaire based on the Delphi Method.

Effectiveness was evaluated by comparing functional capacity and cognitive status outcomes at pre- and immediately post-intervention (with a minimum intervention duration of three consecutive days) or when completing a maximum of nine days of intervention. The functional capacity of participants will be evaluated by the Short Physical Performance Battery (SPPB), the total score ranging from 0 (worst) to 12 points (best) [26] which includes balance, gait, and rising from a chair test. The standing balance test consists of the ability to maintain the standing position for 10 s with three different foot position: parallel, semi-tandem, and tandem. Measurement of walking speed, the time needed to progress for 4 linear meters at the patient’s usual speed, assigning a different score according to the speed. Chair sit-to-stand evaluates the ability to stand from a chair 5 times in a row without using the arms. The SPPB test is considered a valid instrument for detecting frailty and predicting disability, institutionalization, and mortality [27,28].

The cognitive function will be screened by the Mini Mental State Examination (MMSE) test [29], Spanish version [30], which has been widely used to determine cognitive decline. The principle cognitive domains assessed are orientation to time (5 points), orientation to place (5 points), registration of three words (3 points), attention and calculation (5 points), recalling the three words (3 points), language (8 points), and visuospatial ability (1 point). The MMSE is scored out of 30 points, with a score of ≤23 points indicating likely cognitive impairment [31]. The cognitive function will be quantified by the Cambridge Revised Screening Test for the Assessment of Mental Disorders in Old Age (CAMDEX-R) [32], Spanish version [33]. The cognitive domains assessed are: orientation (10 points); language (30 points); memory (27 points); attention and calculation (9 points); praxis (12 point); abstract thinking (8 points); and perception (9 points). The CAMDEX-R is scored out of 105 points English version [32] and 107 points Spanish version [33].

In addition, the ADLs will be assessed using the Barthel Index, Spanish version; scale of 100 [functional independence] to 0 [severe functional dependence]) [34], an international, validated, and the most used tool to measure disability [35]. The mood status will be assessed by the 15-item Yesavage Geriatric Depression Scale; Spanish version; contain ten affirmative items and five negative, a score between 0–5 is normal and a score greater than 5 suggests depression) [36]. The quality of life will be assessed using the quality of life measured by the questionnaires EQ-5D-3L [37]. This instrument measures 5 dimensions of health status: mobility, self-care, usual activities, pain/discomfort, and anxiety/depression. Each dimension is rated according to the following levels: (a) no problems; (b) some problems; (c) extreme problems. Additionally, it contains a visual analogy scale (VAS) to quantify perceived health of 0 [worst health state imaginable] to 100 [best health state imaginable] EuroQol–5 Dimension; Spanish version of the EQ-5D32) [37,38]. Development of delirium will be assessed with the Confusion Assessment Method [39]. Changes in level of physical activity or mobility (step count, walking, and sedentary time per day, will be measured by accelerometer Actigraph^®^ (ActiGraph LLC, Pensacola, Florida, EEUU) [40] and NFC system Health Arcade). Lastly, changes in handgrip strength will be measured using a hand dynamometer (Takei 5401 Digital Dynamometer, Takei scientific instruments CO., LTD, Niigata, Japan) [41]. The maximum value will be recorded after two measurements in each hand. The measurement results are expressed in kilograms [42,43].

### 2.7. Planned Statistical Analysis

We will not report significance tests as the feasibility uncontrolled pre-post design was not designed or powered to test hypotheses or to detect change. A convenience sample size of 40 participants will be selected in accordance with published guidance for feasibility and acceptability of the technology studies [44,45]. According to Hertzog [46], 10–40 subjects provide estimates precise enough to meet a variety of possible aim be considered for pilot studies. In an initial analysis, we will calculate the proportion of enrolled participants out of all eligible patients, participants who adhered to the intervention, and the dropout rates. Besides, a patient satisfaction survey for the use of Health Arcade will be collected and analyzed. Descriptive statistics will be used to summarise markers of feasibility and appeal.

## 3. Expected Results/Discussion

Hospitalization is usually a sentinel event leading to disability, especially in older adults [1,2,3,4]. Although the hospitalization process gives the chance to solve the acute phases of the disease in order to restore the state of health, it can considerably affect the functional capacity of old patients. A growing number of studies are emerging focused on the iatrogenic effects and nosocomial disability developed during hospitalization, and currently these consequences are relevant in clinical practice. The iatrogenic nosocomial disability is mainly characterized by prolonged bed rest episodes, sedentary behaviours and little cognitive stimulation during the entire hospital stay. These low-mobility behaviours coupled with the impaired intrinsic capacity of older people precipitate many adverse effects, such as loss of independence of ADLs, cognitive impairment, reduced caloric intake, sarcopenia, higher risk of falls and social isolation; thus, increasing the likelihood of developing new disabilities and death [4,5,6,7,8,9].

Accordingly, our research team has successfully demonstrated that a multi-component intervention and early physical rehabilitation are a key element in preventing functional and cognitive impairment usually associated with hospitalization in older adults [47,48]. Although the exercise benefits on physical and cognitive function have been researched in hospitalized older medical patients, there is a gap in the research on the role that serious games may have in acute hospital settings among this population [21]. A gamification intervention which includes physical exercise and cognitive training can be an effective strategy to achieve changes in the sedentary behavior of the patients, and that is the reason for developing the validation of a pilot-system multidomain intervention for improving functional abilities during hospital stay. This intervention has been designed focused on the physical and cognitive abilities of the patients, the sociocultural context, and the preferences of older people; as well as the setting in which it is intended to be developed.

## 4. Conclusions

This study’s final outcome is intended to be a robust protocol for a future RCT that will test the effectiveness of a multidomain intervention system based on gamification technologies that will aim to prevent the functional and cognitive decline that usually takes place in hospitalized elderly patients in addition to exploring mechanisms of implementation. This intervention is a potentially valuable benchmark in order to enable high-quality, collaborative discussions and eHealth offers by doctors and other health professionals, along with empowerment for patients to make informed decisions. Thus, if this intervention or “therapeutic decision aid” proves effective, it may set the stage for the development of other decision-making aids that will enable informed choices regarding other geriatric or non-pharmacological approaches, thereby allowing these interventions to be better matched and targeted to what patients actually need or prefer. This knowledge is novel, as there are no pragmatic studies that fills these gaps in the literature based on multi domain gamification strategies. Further consideration of this issue is recommended.

## 5. Trial Status

Recruitment for testing the prototype Health Arcade started on September 2020 and is currently open for recruitment. The research schedule to version 1.0 of the Health Arcade study protocol was affected by the health emergency of COVID-19.

## Figures and Tables

**Table 1:** Eligibility criteria.

Inclusion	Exclusion
Barthel Index score ≥60 points.	Expected length of stay <6 days.
Without major cognitive disorder or with mild cognitive impairment (Mini Mental State Examination ≥19; Global Deterioration Scale score = 1–3).	Uncontrolled arrhythmias, acute pulmonary embolism and myocardial infarction, or extremity bone fracture in the past 3 months.
Able to ambulate (with/without assistance).	Terminal illness.
The participant must be able to communicate and collaborate with the researchers.	

**Table 2:** Content of the training sessions.

Cognitive Area	Name of Game		Description			Sequences		Goals		Total Score
**Day 1**										41
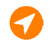	Clock needles		Select the option that corresponds to the time shown on the clock.			6		Select the correct option in less than 20 s.		6
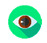	Which figure is it?		Select the option that corresponds to the figure shown on the picture.			6		Select the correct option in less than 15 s.		6
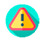	Word soup		Select the option that corresponds to the word hidden in word soup.			6		Select the correct option in less than 20 s.		6
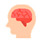	Memorize the picture		Remember and select the option that corresponds to the image shown in the previous image.			6		Select the correct option in less than 15 s.		6
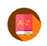	Synonyms		Select the option that corresponds to the synonym of the word that appears on the screen.			6		Select the correct option in less than 20 s.		6
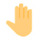	Sequence of functional scopes		Press the button with the number that appears on the screen.			11		Select the correct option in less than 15 s.		11
**Day 2**										41
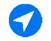	Near or far		Select the option that corresponds to the nearest or farthest object in the picture on the screen.			6		Select the correct option in less than 20 s.		6
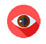	Count the figures		Select the option that corresponds to the total number of figures displayed on the screen.			6		Select the correct option in less than 20 s.		6
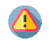	Follow the runner!		Select the option that corresponds to the position that a certain runner has reached.			6		Select the correct option in less than 20 s.		6
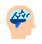	The proverb		Select the option that correctly completes the proverb.			6		Select the correct option in less than 20 s.		6
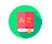	What is it called?		Select the option that corresponds to the utility of the object displayed on the screen.			6		Select the correct option in less than 20 s.		6
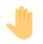	Sequence of functional scopes		Press the button with the number that appears on the screen.			11		Select the correct option in less than 15 s.		11
**Day 3**										41
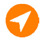	Calendar		Select the option that corresponds to the date shown on the calendar.			6		Select the correct option in less than 20 s.		6
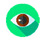	Which figure is it?		Select the option that corresponds to the figure shown on the picture.			6		Select the correct option in less than 15 s.		6
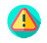	The highest number		Select the option that corresponds to the highest number displayed on the screen.			6		Select the correct option in less than 20 s.		6
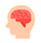	Count the boxes		Remember and select the option that corresponds to the numbers of boxes shown in the previous screen.			6		Select the correct option in less than 20 s.		6
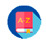	Categories		Select the option that corresponds to the word that is not related to that category of words.			6		Select the correct option in less than 20 s.		6
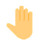	Sequence of functional scopes		Press the button with the number that appears on the screen.			11		Select the correct option in less than 15 s.		11
**Day 4**										41
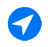	Supermarket shelves		Select the option that corresponds to the position of the food of the picture that appears on the screen.			6		Select the correct option in less than 20 s.		6
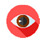	The architect		Select the option that corresponds to the figure shown on the picture.			6		Select the correct option in less than 15 s.		6
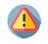	Soup of Symbols		Select the option that corresponds to the number of a certain symbol indicated in each sequence.			6		Select the correct option in less than 20 s.		6
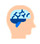	Fruit basket		Select the option that corresponds to the fruits of the picture that appears on the screen.			6		Select the correct option in less than 20 s.		6
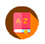	Complete the sentence		Select the option that correctly completes the sentence.			6		Select the correct option in less than 20 s.		6
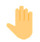	Sequence of functional scopes		Press the button with the number that appears on the screen.			11		Select the correct option in less than 15 s.		11
**Day 5**										41
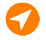	Clock needles		Select the option that corresponds to the time shown on the clock.			6		Select the correct option in less than 20 s.		6
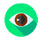	Which figure is it?		Select the option that corresponds to the figure shown on the picture.			6		Select the correct option in less than 15 s.		6
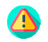	Word soup		Select the option that corresponds to the word hidden in word soup.			6		Select the correct option in less than 20 s.		6
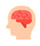	Memorize the picture		Remember and select the option that corresponds to the image shown in the photo previous.			6		Select the correct option in less than 15 s.		6
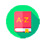	The lost word		Select the option that contains the word that correctly completes the sentence.			6		Select the correct option in less than 20 s.		6
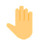	Sequence of functional scopes		Press the button with the number that appears on the screen.			11		Select the correct option in less than 15 s.		11
**Day 6**										41
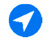	Near or far		Select the option that corresponds to the nearest or farthest object in the picture on the screen.			6		Select the correct option in less than 20 s.		6
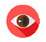	Count the figures		Select the option that corresponds to the total number of figures displayed on the screen.			6		Select the correct option in less than 20 s.		6
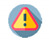	Follow the runner!		Select the option that corresponds to the position that a certain runner has reached.			6		Select the correct option in less than 20 s.		6
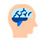	The proverb		Select the option that correctly completes the proverb.			6		Select the correct option in less than 20 s.		6
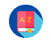	What would you take with you?		Select the option that contains the most appropriate object for the proposed scenario or situation.			6		Select the correct option in less than 20 s.		6
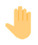	Sequence of functional scopes		Press the button with the number that appears on the screen.			11		Select the correct option in less than 15 s.		11
**Day 7**										41
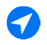	Calendar		Select the option that corresponds to the date shown on the calendar.			6		Select the correct option in less than 20 s.		6
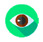	Which figure is it?		Select the option that corresponds to the figure shown on the picture.			6		Select the correct option in less than 15 s.		6
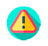	The highest number		Select the option that corresponds to the highest number displayed on the screen.			6		Select the correct option in less than 20 s.		6
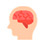	Count the boxes		Remember and select the option that corresponds to the numbers of boxes shown in the previous screen.			6		Select the correct option in less than 20 s.		6
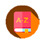	Situations		Select the option that corresponds to the action or activity shown in the image.			6		Select the correct option in less than 20 s.		6
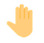	Sequence of functional scopes		Press the button with the number that appears on the screen.			11		Select the correct option in less than 15 s.		11
**Day 8**										41
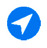	Supermarket shelves		Select the option that corresponds to the position of the food of the picture that appears on the screen.			6		Select the correct option in less than 20 s.		6
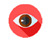	The architect		Select the option that corresponds to the figure shown in the picture.			6		Select the correct option in less than 15 s.		6
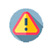	Soup of Symbols		Select the option that corresponds to the number of a certain symbol indicated in each sequence.			6		Select the correct option in less than 20 s.		6
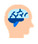	Fruit basket		Select the option that corresponds to the fruits of the picture that appears on the screen.			6		Select the correct option in less than 20 s.		6
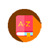	Categories		Select the option that corresponds to the word that does not correspond to that category of words.			6		Select the correct option in less than 20 s.		6
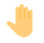	Sequence of functional scopes		Press the button with the number that appears on the screen.			11		Select the correct option in less than 15 s.		11
**Day 9**										41
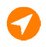	Clock needles		Select the option that corresponds to the time shown in the clock.			6		Select the correct option in less than 20 s.		6
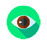	Which figure is it?		Select the option that corresponds to the figure shown in the picture.			6		Select the correct option in less than 15 s.		6
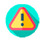	Word soup		Select the option that corresponds to the word hidden in word soup.			6		Select the correct option in less than 20 s.		6
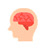	Memorize the picture		Remember and select the option that corresponds to the image shown in the previous image.			6		Select the correct option in less than 15 s.		6
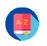	Ferris wheel of letters		Select the word that can be formed from the letters that appear on the screen.			6		Select the correct option in less than 20 s.		6
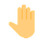	Sequence of functional scopes		Press the button with the number that appears on the screen.			11		Select the correct option in less than 15 s.		11
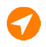		Temporal orientation		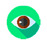	Visual perception		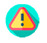		Selective attention	
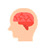		Short-term, operational and working memory		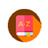	Linguistic expression and understanding		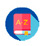		Categorization and semantic awareness	
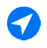		Spatial orientation		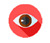	Spatial perception		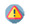		Sustained attention	
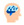		Semantic memory		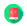	Denomination		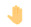		Oculo-manual coordination control	
